# Long-Term Effects of Home-Based Bench-Stepping Exercise Training on Healthcare Expenditure for Elderly Japanese

**DOI:** 10.2188/jea.JE20100103

**Published:** 2011-09-05

**Authors:** Yukari Mori, Takuro Tobina, Koji Shirasaya, Akira Kiyonaga, Munehiro Shindo, Hiroaki Tanaka

**Affiliations:** 1 Graduate School of Sports and Health Science, Fukuoka University, Fukuoka, Japan; 2 Faculty of Sports Science, Fukuoka University, Fukuoka, Japan; 3 Faculty of Economics, Fukuoka University, Fukuoka, Japan; 4 Institute for Physical Activity, Fukuoka University, Fukuoka, Japan

**Keywords:** aerobic capacity, health promotion, number of outpatient visits, outpatient expenditure

## Abstract

**Background:**

We examined the long-term effects of home-based bench-stepping exercise training on total healthcare expenditure (TOHEX) and number of outpatient visits (NOVIS) in elderly adults.

**Methods:**

A total of 189 elderly Japanese (age 73 ± 4 years) participated in this study. They were randomly assigned to either an exercise or control group. TOHEX, NOVIS, and outpatient expenditure (OPEX) were evaluated every 6 months from 1 year before the start to the end of the intervention period, as well as 1 year after the end of the intervention. The exercise group was encouraged to perform home-based bench-stepping exercise training on most, and preferably all, days of the week for 18 months.

**Results:**

The exercise group showed significant increases in lactate threshold as compared with pre-intervention values. There were no significant differences in TOHEX, OPEX, or NOVIS between the exercise and control groups 1 year before the start of the intervention, and the values remained similar during the first 12 months of the intervention period. However, at 18 months, TOHEX, NOVIS, and OPEX were significantly lower in the exercise group than in the control group (TOHEX: 170 007 ± 192 072 vs. 294 705 ± 432 314 yen, *P* = 0.008; NOVIS: 19.2 ± 26.3 vs. 28.2 ± 32.1 days, *P* = 0.012; OPEX: 132 973 ± 132 016 vs. 187 799 ± 158 167 yen, *P* = 0.005).

**Conclusions:**

The data indicate that a long-term home-based bench-stepping exercise program can reduce healthcare expenditure in elderly Japanese.

## INTRODUCTION

Expenditures on healthcare and long-term care have continued to increase in Japan as the population ages.^1^ Senior citizens (adults older than 65 years) comprised 22% of the population in 2009,^2^ and this percentage is continuing to increase. Measures to prevent lifestyle-related diseases and age-related muscle degeneration are needed to maintain quality of life in this segment of the community. Previous studies have determined that moderate-intensity exercise training improves hypertension and glucose and lipid metabolism.^3^^–^^7^ These effects on the cardiovascular system and metabolism might help to maintain quality of life and reduce healthcare expenditure and nursing-care expenses.

It has been suggested that lower aerobic capacity (V^·^O_2_ max) is a risk factor for increased healthcare expenditure.^8^ Kawaguchi et al reported that healthcare expenditure was associated with body mass index (BMI) and daily walking distance.^9^ However, little is known regarding the effects of exercise-training interventions on healthcare expenditure. Kawaguchi et al also reported that combined aerobic and resistance training appeared to reduce healthcare expenditure as compared with a control group.^9^ However, that study was limited by its small sample size and significant differences in average age and baseline healthcare expenditure between groups. The present study therefore aimed to evaluate the effects of an exercise-training intervention on total healthcare expenditure (TOHEX), number of outpatient visits (NOVIS), and outpatient expenditure (OPEX) in a randomized trial with a larger sample size.

## METHODS

### Subjects

This study was carried out in Neagari-machi in Ishikawa prefecture, Japan. A total of 220 people were randomly selected from the local resident registry and were contacted by telephone. Of these, 211 (96%) attended a study information session held at the Neagari-machi Welfare Center in May 2003. A total of 189 people (86% of the original 220) subsequently entered the study (mean age: 73 ± 4 years; 104 men and 85 women). All subjects received a medical check-up that included electrocardiography and clinical history-taking.

The subjects were randomly assigned to either the exercise group or the control group. The exercise group included 98 subjects (aged 73 ± 4 years; 54 men and 44 women), and the control group included 91 subjects (aged 73 ± 4 years; 50 men and 41 women). The characteristics of the subjects are shown in Table 1.

**Table 1.** Characteristics of study subjects

	Exercise group *n* = 98	Control group *n* = 91	*P* value
Male/female	54/44	50/41	0.463
Age (years)	73 ± 4	73 ± 4	0.871
Height (cm)	155.9 ± 8.7	154.4 ± 9.3	0.281
Weight (kg)	57.6 ± 10.5	55.8 ± 9.2	0.202
BMI (kg/m^2^)	23.6 ± 3.4	23.3 ± 3.1	0.525

Abbreviation: BMI, body mass index.

Data are shown as mean ± standard deviation.

Characteristics were measured in May 2003. The male/female ratio was similar in the exercise and control groups (χ^2^ test). Characteristics were compared using the unpaired Student *t*-test. There were no differences between groups.

Forty-eight subjects in the exercise group received no medication in May 2003. The remaining 50 subjects received medication for hypertension (*n* = 35), vascular diseases (*n* = 18), gastrointestinal disorders (*n* = 15), hyperlipidemia (*n* = 12), sleep-inducing drugs and antidepressants (*n* = 8), diabetes mellitus (*n* = 5), osteoporosis (*n* = 4), thyroid disorders (*n* = 2), anemia (*n* = 1), and/or postmenopausal syndrome (*n* = 1).

The experimental procedure was approved by the ethics committee of Fukuoka University. Written informed consent was obtained from all subjects.

### Assessment of TOHEX, NOVIS, and OPEX

Information on medical claims was obtained from the Neagari Municipal Health Insurance office. TOHEX, NOVIS, and OPEX were evaluated by examining monthly health insurance claims every 6 months from before the start to the end of the intervention period. Data were collected at 1 year and at 6 months before starting the intervention, which are designated as the first (June 2002 to November 2002) and second (December 2002 to May 2003) periods, respectively. Data were also collected every 6 months during the intervention period for 18 months, and these are designated as the third (June 2003 to November 2003), fourth (December 2003 to May 2004), and fifth (June 2004 to May 2004) periods. In addition, data were collected 1 year after the end of intervention, which is designated as the sixth period (June 2005 to November 2005).

To compare changes in TOHEX, NOVIS, and OPEX in subjects requiring medical treatment, only subjects who received medical consultations were included in the analyses. Thus, TOHEX, NOVIS, and OPEX were defined in this study as the mean values for subjects who received medical consultations. Four subjects in the exercise group and 6 in the control group received no medication during the intervention period and were therefore excluded from the analyses of TOHEX, NOVIS, and OPEX. In addition, 14 subjects in the exercise group and 8 in the control group were excluded during the first period, as well as 12 and 6 during the second, 10 and 6 during the third, 10 and 4 during the fourth, and 11 and 9 during the fifth, respectively. In the sixth period, 29 and 19 subjects in the exercise and control groups, respectively, were excluded because they did not receive any medication, had moved, or had died. Thus, the subjects included in the analyses of TOHEX, NOVIS, and OPEX received similar medical services during the first to the sixth period and were followed-up until the sixth period.

### Distribution of TOHEX

Subjects were categorized according to TOHEX, and the distribution of TOHEX values was compared between groups. The lowest average TOHEX value was 170 007 yen (fifth period in the exercise group). A TOHEX less than 150 000 yen was therefore defined as low TOHEX, 150 000 to less than 300 000 yen was defined as moderate TOHEX, and 300 000 yen or more was defined as high TOHEX. The numbers of subjects were counted, and the ratios were calculated.

### Assessment of aerobic capacity, leg extension power, and blood pressure

Lactate threshold (LT, an index of aerobic capacity), leg extension power (LEP), and blood pressure were measured in the exercise group at 0 months (May 2003), 6 weeks (July 2003), 3 months (August 2003), and 18 months (August 2005). A submaximal graded bench-stepping test was conducted to determine individualized LT. The experiment determined the exercise intensity equivalent to the LT, which was designated as metabolic equivalents (METs) at LT. The METs value was calculated from the step height and the number of ascents per minute by the formula described in the American College of Sports Medicine guidelines for exercise tests and prescription.^10^ This procedure has been described previously.^11^

LEP was evaluated using an isodynamometer (Anaero Press 3000; Combi Co. Ltd.; Tokyo, Japan). Subjects sat on the seat and put their legs on the kicking platform. They practiced kicking twice slowly, and the length of their legs was measured. Finally, the subjects pushed out the plate 5 times as quickly as possible. The mean of the maximal and second maximal values was normalized by body weight to determine LEP.^11^^,^^12^

Blood pressure was measured twice after resting using a digital automatic blood pressure meter (HEM-705IT Fuzzy; Omron; Tokyo, Japan). If the systolic blood pressure (SBP) or diastolic blood pressure (DBP) differed by more than 10 mm Hg between the first and second test, then a third test was carried out. The mean of 2 or 3 values was used as the blood pressure value. All blood pressure measurements were carried out between 8:30 AM and noon.^12^

### Exercise program

The exercise intervention program started in May 2003. Subjects in the exercise group were encouraged to perform home-based bench-stepping exercise training on most, and preferably all, days of the week for 18 months. Exercise intensity was prescribed in accordance with the height of the bench step and the frequency of ascents and descents corresponding to the individualized LT, which was readjusted after the first 6 weeks. The exercise group was also scheduled to attend group exercise sessions throughout the 18-month intervention period. The group exercise sessions were conducted weekly at a community health center during the initial 3 months of the intervention and then biweekly for the remainder of the study period.

The amount of exercise performed by each participant in the exercise group, including the time spent on exercise, was noted daily in an exercise-training log, and the weekly sum was determined during the 18-month intervention period.

### Statistical analysis

All data are expressed as mean values ± standard deviations. The male/female ratios in the exercise and control groups were compared using the χ^2^ test. The characteristics were compared using the unpaired Student *t*-test. One-factor repeated-measures analysis of variance was used to compare any within-group changes in weight, BMI, SBP, DBP, LEP, and LT in the exercise group. The Tukey-Kramer test was used as a post-hoc test for comparisons between periods within groups. The Mann-Whitney U-test was used to compare TOHEX, NOVIS, and OPEX between groups for each period. The distributions of TOHEX were compared between the exercise and control groups using the χ^2^ test. In addition, odds ratios were calculated in the exercise group using logistic regression analysis based on the number of subjects, categorized by their TOHEX.

Statistical analysis was carried out using SPSS 14.0J for Windows (SPSS, Cary, NC, USA). In all instances, a *P* value less than 0.05 was considered to indicate statistical significance.

## RESULTS

The male/female ratio was similar in both groups. Seventy-five participants in the exercise group completed 3 months of the exercise-training program. They conducted bench-stepping exercise training for an average of 179 ± 43 minutes every week during the initial 3 months of the intervention, resulting in a significant increase in METs at LT as compared with baseline (Table 2). Forty-three of 98 subjects continued to perform the training for an average of 178 ± 47 minutes every week throughout the 18-month intervention period. METs at LT were maintained at the same level after 3 months of training in these 43 subjects (Table 2).

**Table 2.** Changes in physiological variables during the 18 months of the intervention in the exercise group

	0 months May 2003	6 weeks July 2003	3 months August 2003	*P* value		18 months August 2005	*P* value
	
Weight (kg)	58.0 ± 10.1	57.8 ± 10.1	57.5 ± 9.8^a^	0.009		59.6 ± 10.2^c^	0.022
BMI (kg/m^2^)	23.7 ± 3.3	23.6 ± 3.3	23.5 ± 3.2^a^	0.011		24.0 ± 3.4	0.031
SBP (mm Hg)	133 ± 19	128 ± 17^a^	127 ± 17^a^	0.012		133 ± 17	0.121
DBP (mm Hg)	72 ± 9	70 ± 10	68 ± 9^a^	0.004		75 ± 9^c^	<0.001
LEP (watts/kg)	8.2 ± 4.9	8.6 ± 4.9	10.0 ± 4.5^a,b^	<0.001		8.8 ± 3.2^c^	<0.001
LT (METs)	4.2 ± 0.8	4.6 ± 0.8^a^	5.0 ± 0.7^a,b^	<0.001		4.9 ± 1.0^a^	<0.001

Abbreviations: BMI, body mass index; SBP, systolic blood pressure; DBP, diastolic blood pressure; LEP, leg extension power; LT, lactate threshold; METs, metabolic equivalents.

Data are shown as mean ± standard deviation.

The 75 subjects in the exercise group (44 men and 31 women) who completed the 3-month intervention were included in the longitudinal analysis for 0–3 months. The 43 subjects (25 men and 18 women) who completed the 18-month intervention were included in the longitudinal analysis for 0–18 months. One-factor repeated-measures analysis of variance was used to compare any within-group changes in parameters. The results are shown with the *P* values. The Tukey-Kramer test was used as a post-hoc test.

^a^*P* < 0.05 vs. 0 months; ^b^*P* < 0.05 vs. 6 weeks; ^c^*P* < 0.05 vs. 3 months.

Weight and BMI were significantly lower after 3 months of intervention; however, weight was higher at 18 months than at 3 months. SBP decreased rapidly during the exercise intervention, and DBP was lower after 3 months of intervention. However, these effects had disappeared at 18 months. Although LEP was significantly higher at 3 months, it significantly decreased between 3 months and 18 months.

Information on medical claims was obtained from all subjects who had medical consultations. A total of 10 366 medical claims were made, 96% of which were accounted for by 10 specialties: internal medicine, 40.8%; orthopedic surgery, 12.6%; ophthalmology, 10.0%; dispensing pharmacy, 6.5%; dentistry, 6.3%; urology, 5.7%; dermatology, 4.2%; cerebral surgery, 4.1%; surgery, 3.7%; and otorhinolaryngology, 1.9%.

There were no differences between groups in TOHEX, NOVIS, or OPEX during the first to fourth periods; however, these values were significantly lower in the exercise group as compared with the control group during the fifth period (Tables 3–5). There were no significant differences in TOHEX, NOVIS, or OPEX between groups in the sixth period.

**Table 3.** Effect of long-term bench-stepping exercise training on total healthcare expenditure (yen)

Period			Exercise group			Control group			*P* value
	
			*n*	Mean	SD	*n*	Mean	SD	
Before intervention	1st	June 2002 November 2002	80	232 640	358 232	77	280 116	719 293	0.969
	2nd	December 2002 May 2003	82	197 789	328 821	79	185 565	242 365	0.908

Intervention	3rd	June 2003 November 2003	84	299 777	610 750	79	301 394	339 693	0.219
	4th	December 2003 May 2004	84	228 713	344 739	78	312 777	463 134	0.103
	5th	June 2004 November 2004	83	170 007	192 072	76	294 705	432 514	0.008

1 year after intervention	6th	June 2005 November 2005	63	313 744	500 569	66	395 133	644 380	0.132

The Mann-Whitney U-test was used to compare total healthcare expenditures between groups during each period. A significant difference was observed in the fifth period.

**Table 4.** Effect of long-term bench-stepping exercise training on number of outpatient visits

Period			Exercise group			Control group			*P* value
	
			*n*	Mean	SD	*n*	Mean	SD	
Before intervention	1st	June 2002 November 2002	80	28.2	31.2	77	22.5	21.0	0.240
	2nd	December 2002 May 2003	82	21.1	23.6	79	17.4	14.1	0.688

Intervention	3rd	June 2003 November 2003	84	23.9	28.1	79	26.5	27.2	0.403
	4th	December 2003 May 2004	84	22.0	26.2	78	24.1	24.2	0.330
	5th	June 2004 November 2004	83	19.2	26.3	75	28.2	32.1	0.012

1 year after intervention	6th	June 2005 November 2005	63	28.3	35.1	66	28.4	26.8	0.754

The Mann-Whitney U-test was used to compare number of outpatient visits during each period. A significant difference was observed in the fifth period.

**Table 5.** Effect of long-term bench-stepping exercise training on outpatient expenditure (yen)

Period			Exercise group			Control group			*P* value
	
			*n*	Mean	SD	*n*	Mean	SD	
Before intervention	1st	June 2002 November 2002	80	162 033	112 410	77	162 150	124 303	0.661
	2nd	December 2002 May 2003	82	131 632	96 927	79	138 143	105 247	0.738

Intervention	3rd	June 2003 November 2003	84	154 837	106 001	79	194 652	135 958	0.092
	4th	December 2003 May 2004	84	145 612	104 393	78	185 031	152 160	0.107
	5th	June 2004 November 2004	83	132 973	132 016	75	187 799	158 167	0.005

1 year after intervention	6th	June 2005 November 2005	63	178 033	143 398	66	225 643	206 403	0.144

The Mann-Whitney U-test was used to compare outpatient expenditures between groups during each period. A significant difference was observed in the fifth period.

The distribution of TOHEX values is shown in the Figure. The distributions differed significantly between the exercise and control groups in the third, fourth, and fifth periods, but not in the sixth period (*P* = 0.059). The probability of TOHEX exceeding 300 000 yen was lower in the exercise group than in the control group in the fourth and fifth periods, but not in the third period (*P* = 0.061) (Table 6).

**Figure.**
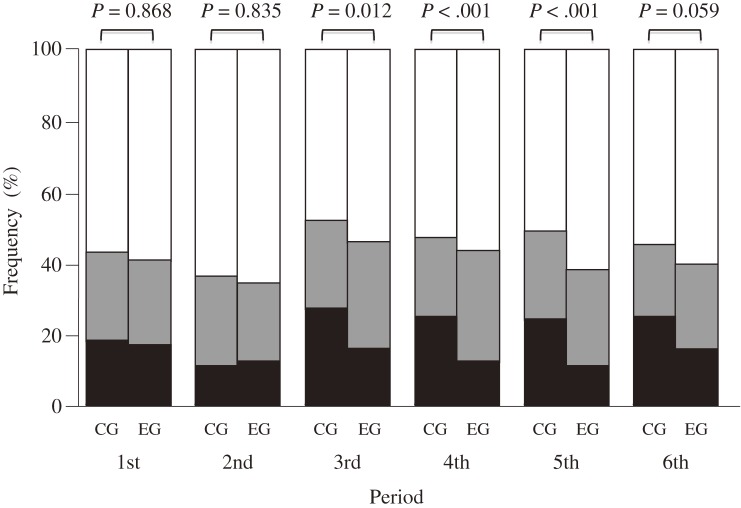
Distribution of subjects by total healthcare expenditure. The bars indicate the proportions of TOHEX values that were less than 150 000 yen (white), 150 000 to less than 300 000 yen (gray), and 300 000 yen or more (black). The distributions were compared by using the χ^2^ test. Abbreviations: CG, control group; EG, exercise group; TOHEX, total healthcare expenditure.

**Table 6.** Odds ratios for total healthcare expenditures in the exercise group, as compared with the control group, before, during, and after exercise

Period		<150 000 yen	150 000– <300 000 yen	≥300 000 yen
Before intervention	1st	1.12 (0.62–2.03)	0.93 (0.47–1.84)	0.91 (0.42–1.98)
	2nd	1.07 (0.58–1.98)	0.87 (0.43–1.74)	1.10 (0.44–2.74)

Intervention	3rd	1.28 (0.71–2.31)	1.31 (0.67–2.53)	0.50 (0.24–1.04)^c^
	4th	1.12 (0.62–2.02)	1.67 (0.85–3.28)	0.42 (0.19–0.94)^a^
	5th	1.55 (0.85–2.81)	1.11 (0.57–2.17)	0.40 (0.18–0.92)^b^

1 year after intervention	6th	1.29 (0.71–2.34)	1.20 (0.59–2.45)	0.56 (0.27–1.18)

Data are shown as odds ratios and 95% confidence intervals. The odds ratios for expenditure of ≥300 000 yen were significantly lower in the exercise group as compared with the control group in the fourth (^a^*P* = 0.031) and fifth periods (^b^*P* = 0.027), but not in the third period (^c^*P* = 0.061).

## DISCUSSION

The main finding of this study was that long-term home-based bench-stepping exercise training reduced TOHEX, NOVIS, and OPEX as compared with a control group with no exercise intervention. A previous study reported that short-term (3 months) home-based bench-stepping exercise training led to large increases in LT and LEP in older adults.^11^ The participants in our study also showed improvements in LT after 3 months.

Healthcare expenditure is affected by numerous factors, including age, personal medical history, lifestyle habits, and proximity to a medical institution.^9^ A single individual with an unplanned critical illness could affect the results of the analysis of a small sample. Little is known regarding the effects of exercise-training intervention on healthcare expenditure. Kawaguchi et al reported that a combined aerobic and resistance-training intervention program appeared to reduce healthcare expenditure in a small sample as compared with a control group.^9^ The present study used a randomized trial design and a larger sample population and was thus able to more accurately demonstrate the effects of exercise intervention on healthcare expenditure.

Half of the medical claims in this study were accounted for by internal medicine or orthopedic surgery. In the exercise group, 45 of 98 (46%) subjects received medications related to internal medicine, suggesting that prevention of such diseases could significantly reduce healthcare expenditure. Previous studies have demonstrated that exercise training of moderate intensity is beneficial for the prevention and treatment of illness. The current results showed that SBP and DBP were significantly lower at 3 months of exercise intervention. Our previous study showed that LT-intensity exercise training decreased blood pressure to normotensive levels in hypertensive patients via reductions in blood catecholamine concentrations.^3^ Exercise also induces nitric oxide production, which contributes to the reduction of blood pressure in hypertensive patients.^13^ In addition, LT-intensity exercise training improves glucose and insulin sensitivity and high-density lipoprotein cholesterol levels.^7^^,^^8^ In the present study, weight and BMI were lower in the exercise group after 3 months, which could also contribute to improvements in hypertension and both glucose and lipid metabolism. Furthermore, our study confirmed that bench-stepping exercise training significantly improved leg strength in elderly adults. A care report showed that frail subjects were able to walk without a cane after 3 months of bench-stepping exercise. The present results indicate that bench-stepping exercise training can prevent age-related muscle degeneration and thus help to reduce healthcare expenditure.

In the current study, TOHEX, NOVIS, and OPEX were significantly lower in the exercise group than in the control group in the fifth period, which suggests that adaptation of aerobic capacity, blood pressure, and glucose and lipid metabolism occurred relatively early, while it took longer to reduce TOHEX, NOVIS, and OPEX. The values of OPEX divided by NOVIS were 6926 and 6659 yen in the exercise and control groups, respectively. The similarity of these values indicates that the reduction in OPEX depended on NOVIS. The medical treatment received at each outpatient visit was similar in both groups; however, the overall frequency of visits was lower in the exercise group. Although the reductions in weight, BMI, SBP, and DBP, and improvements in LEP had disappeared by 18 months, LT remained higher at that time point. It is possible that improved physical fitness and/or the exercise intervention itself reduced subjects’ anxiety about their health and that these physical and mental changes contributed to reductions in NOVIS.

TOHEX distributions significantly differed in the third, fourth, and fifth periods, with a trend in the sixth period. The probability of a TOHEX greater than 300 000 yen was significantly lower in the exercise group in the third, fourth, and fifth periods, as compared with the control group. These results suggest that exercise training can prevent increases in TOHEX in subjects with relatively severe diseases and support the hypothesis that maintaining high aerobic capacity prevents an increase in healthcare expenditure.^8^

TOHEX, NOVIS, and OPEX in the second period were lower than in the first period in both the exercise and control groups. However, the slight decreases seen in both groups during the second period might reflect the fact that the calculation of healthcare expenditure was revised in October 2002, and insurance for elderly people was decreased. Although healthcare expenditure is influenced by many factors, including legislation, it gradually decreased in the exercise group in the third, fourth, and fifth periods (by 71 064 yen from the third to the fourth periods and by 58 706 yen from the fourth to the fifth periods). However, in the control group it increased by 11 383 yen from the third to the fourth periods and decreased by 18 072 yen from the fourth to the fifth periods. These results indicate that exercise training may reduce healthcare expenditure.

This study has some limitations. First, the claims were not clearly classified by disease, and the study was therefore unable to determine which types of healthcare expenditures were affected by exercise training. Second, the exercise intervention may have inspired subjects in the exercise group to adopt other health-promoting behaviors in addition to exercise. Third, measurements of LT and LEP, and other medical examinations, were not continually performed in the control group. Although we found a substantial reduction in TOHEX, NOVIS, and OPEX in the exercise group as compared with the control group, further follow-up results are required to determine if these trends continue over a longer period of time.

In conclusion, a long-term home-based bench-stepping exercise program can lead to substantial reductions in healthcare expenditure associated with the care of elderly adults.
